# Platelet derivatives in oral and maxillofacial surgery: classification, and clinical applications

**DOI:** 10.3389/fcell.2026.1776538

**Published:** 2026-02-24

**Authors:** Antonio Scarano, Sergio Alexandre Gehrke, Ermal Pashaj, Gianluca Nicolai, Luan Mavriqi, Edit Xhajanka, Sergio Rexhep Tari

**Affiliations:** 1 Department of Innovative Technologies in Medicine and Dentistry, University of Chieti-Pescara, Chieti, Italy; 2 Department of Research, Bioface/PgO/UCAM, Montevideo, Uruguay, Department of Biotechnology, Univer-sidad Católica de Murcia (UCAM), Murcia, Spain; 3 Department of Surgery, Catholic University Our Lady of Good Council, Tirana, Albania; 4 Department of Maxillo-Facial Surgery, University of Tor Vergata, Rome, Italy; 5 Department of Dental Medicine, Medical University of Tirana, Tirana, Albania

**Keywords:** growth factors, oral maxillofacial surgery, platelet concentrates, platelet-rich plasma, tissue regeneration

## Abstract

Several studies on the use of platelet concentrates show different results. For example, while many studies demonstrate significant benefits of using PCs in tissue regeneration, others report modest or no results, creating uncertainty about the actual clinical usefulness of these PCs. This confusion also arises from the different protocols used to prepare platelet concentrates, which became clearer after the classification of PCs based on the presence of leukocytes and the organization of fibrin. Currently, we have numerous devices available to obtain PCs with specific characteristics typical of each method, such as APG, PRF, PRGF, CGF, *etc.* On one hand, this wide range of options offered by companies creates confusion; on the other hand, it allows us to have systems that provide a uniform product for all operators by standardizing centrifugal force, the type of vial used, its inclination, and rotation time. The aim of this mini review is to provide an overview of the applications of Autologous Platelet Concentrates (APCs) and their clinical application.

## Graphical Abstract

**Figure:**
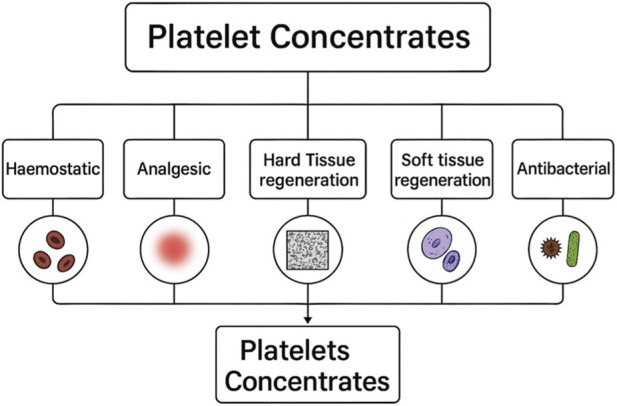

## 1 Introduction

Platelet derivatives represent a rich source of growth factors, including platelet-derived growth factor (PDGF), transforming growth factor beta (TGF-β), and vascular endothelial growth factor (VEGF), which are essential for osteogenesis and angiogenesis (Burnouf et al., 2013). The term “Platelet-rich plasma” was at first introduced by Marx et al. (1998) after which it became widely adopted in the literature. The first generation of platelet concentrates, represented by PRP, is obtained by centrifuging whole blood with the addition of anticoagulants and subsequent activators (Marx et al., 1998). However, the use of chemical additives in this process may interfere with the healing process (Miron and Zhang, 2018).

The second generation, PRF, eliminates anticoagulants, allowing the blood to clot naturally and forming a three-dimensional fibrin matrix (Shah et al., 2017). More recently, third-generation protocols based on low-speed centrifugation (LSCC), such as advanced PRF (A-PRF) and injectable PRF (i-PRF), have been developed. Recent studies highlight conflicting results in bone regeneration, and we also observe numerous preparation protocols referred to by different authors as PRF, PRP, CGF, PRFG, *etc.*

Due to the variability in preparation protocols used, some authors have been concerned with standardizing the quality of the product obtained. PRP is better characterized based on three parameters that take into account platelet concentration compared to baseline, platelet activation method, and total white blood cell (WBC) content, with particular attention to the inclusion or exclusion of neutrophils (DeLong et al., 2012).

This approach led to the development of the PAW classification system (Platelets, Activation, and White blood cells), which provides a more quantitative assessment of the cellular components present (DeLong et al., 2012). Another classification is called PLRA and based on the on four key variables: platelet concentration, presence or absence of leukocytes, red blood cells (RBC), and activation method (Mautner et al., 2015). Subsequently, in 2016, Magalon et al. proposed the DEPA classification system, which considers the quality of PRP preparation through four key factors: dose, efficiency, purity, and activation, thus providing a comprehensive framework for evaluating both the composition and bioactivity of PRP products (Magalon et al., 2016).

The aim of this mini review is to provide an overview of the applications of Autologous Platelet Concentrates (APCs) and their clinical application.

## 2 Clinical applications in oral and maxillofacial surgery

### 2.1 Post-extraction socket preservation

Platelet concentrates are used to minimize bone resorption after extraction (Thakre and Bajaj, 2025). When used alone or combined with bone grafts, platelet concentrates significantly reduce postoperative pain and edema, improving soft tissue healing in the early days. Platelets concentrate are principally used in post-extraction sites to enhance wound healing, reduce complications, and promote tissue regeneration. Platelet concentrates significantly improved soft tissue healing and reduced the incidence of alveolar osteitis (AO). However, their impact on hard tissue healing remains controversial, and future studies should explore alternative methods to assess the effects of platelet concentrates on bone healing and volume maintenance (Hajibagheri et al., 2025). Although some studies show no long-term advantage in preserving volume compared to spontaneous healing, PCs enhance the density and quality of regenerated bone (Shah and Cairns, 2018).

### 2.2 Sinus lifting

PCs are used to fill the space between the sinus membrane and the residual bone in the maxillary sinus lift technique. They are often mixed with autologous bone or biomaterials to form the so-called sticky bone or bone graft (Zhao et al., 2025). When used with the transcrestal approach, it is preferable to use them without biomaterials to avoid perforating the membrane.

They can serve as the sole grafting material (in transcrestal or lateral window techniques) or be mixed with xenogenic materials to create the so-called “sticky bone.” The combination with PCs accelerates the formation of new bone and reduces the time required for implant osseointegration (Malcangi et al., 2023). Platelet concentrates may reduce the maturation time of the bone graft, allowing earlier implant placement, but they do not improve long-term bone formation.

### 2.3 Periodontal and endodontic regeneration

PC are effective in the treatment of intrabony defects and furcation lesions, leading to greater reduction in probing depth and clinical attachment gain (Panda et al., 2019). PCs act as reservoirs for growth factors and cytokines, functioning as a biological scaffold that supports the migration and proliferation of cells necessary for periodontal regeneration (Varshney et al., 2023). A review demonstrated a positive effect in treating permanent teeth with root development, with thickening and elongation of root walls and closure of the apical foramen (Metlerska et al., 2019).

## 3 Discussion

**FIGURE 1:**
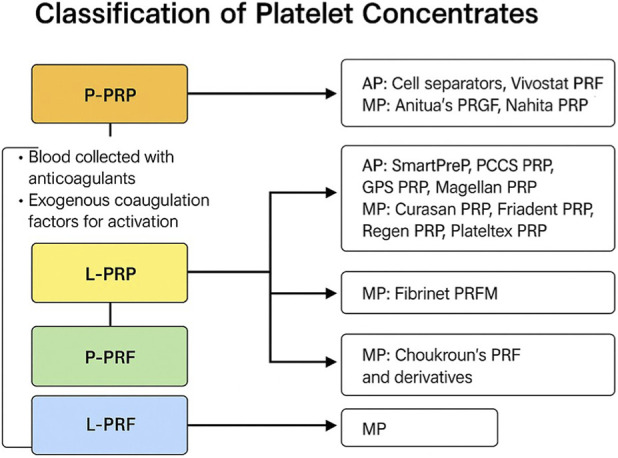
The flowchart illustrates the main categories of autologous platelet concentrates, including P-PRP (Pure Platelet-Rich Plasma), L-PRP (Leukocyte- and Platelet-Rich Plasma), P-PRF (Pure Platelet-Rich Fibrin), and L-PRF (Leukocyte- and Platelet-Rich Fibrin). Each category is associated with its preparation characteristics (e.g., use of anticoagulants, activation pathway) and examples of commercial systems (AP: automated processes; MP: manual processes).

Platelet concentrates were initially used for the treatment of patients suffering from severe thrombopenia, and the first studies, due to a lack of an organized terminology, referred to them generically such as platelet -fibrinogen-thrombine mixture (Rosenthal et al., 1975), platelet-derived wound healing factors (PDWHF) (Knighton et al., 1986), or gelatine platelet (Alidadi et al., 2017). When we talk about Platelet Concentrates (PCs), we should primarily refer to the classification proposed by Dohan et al. (2009), which considers the presence of leukocytes and the organization of the fibrin matrix (liquid or solid). Based on this, we have: Pure Platelet-Rich Plasma (P-PRP) (low leukocytes, liquid/gel), Leukocyte-Platelet-Rich Plasma (L-PRP) (high leukocytes, liquid/gel), Pure Platelet-Rich Fibrin (P-PRF) (no leukocytes, dense fibrin matrix), and Leukocyte-Platelet-Rich Fibrin (L-PRF) (high leukocytes, dense fibrin matrix) (Figure 1). Therefore, we essentially have four families of platelet concentrates. In dentistry, several preparation protocols for PCs have been developed, considering that variables such as centrifugal force (CF) and the type of tube used influence the final product (Figure 1). In dentistry and maxillofacial surgery, PRF is widely used because it also serves as a scaffold and filler. Platelet concentrates (PCs) are employed because, once applied to the bone defect, they provide a quantity of growth factors such as PDGF, IGF, EGF, and VEGF at a concentration higher than what platelets arriving from blood could offer (Egierska et al., 2023). These growth factors promote cell migration, proliferation, and differentiation, mechanisms that are considered the basis of their positive effect on tissue regeneration.

The growth factors contained in PCs act like “conductors,” recruiting the cells responsible for tissue regeneration. The presence of leukocytes, once considered undesirable due to the risk of triggering inflammation, is now believed to play an important role in bone healing, particularly in reducing infections through immune system regulation (Anitua et al., 2021). They also contribute to pain reduction and promote neoangiogenesis (Blanco et al., 2000).

**FIGURE 2:**
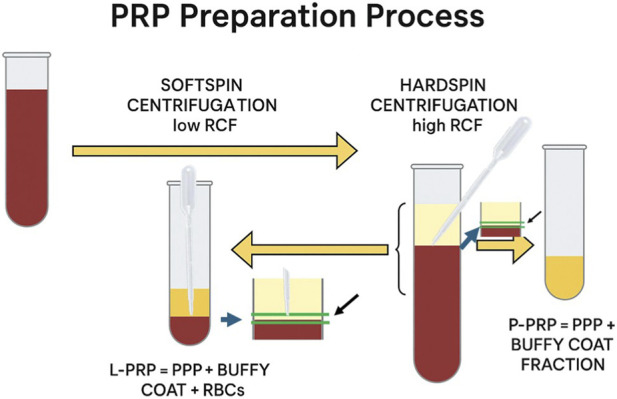
The image shows the steps involved in preparing the platelet concentrate and the pipette collection of the thin layer of platelet-rich plasma and leukocytes located at the interface with the red blood cell layer. The double centrifugation method makes it easier and more likely to collect the buffy coat and platelet-rich plasma.

This naturally raises the question: how do we produce PRP with or without leukocytes? Technology provides us with manual and automated systems that help minimize production errors. Manual systems use centrifuges to separate the blood components (Figure 2). For tubes that generate PRF, three main layers can be identified: red blood cells at the bottom, followed by a solid fibrin architecture that traps platelets and leukocytes, and finally platelet-poor plasma (PPP) at the top.

In the case of PRP, where the fibrin is liquid, it is difficult to accurately collect the platelet concentrate with or without the buffy coat (which contains leukocytes) using pipetting (Figure 2). Typically, in manual procedures, this is done by visual inspection, the operator, based on experience, identifies the whitish layer containing white blood cells (Figure 2). Obviously, this procedure is subject to individual error depending on the operator’s level of expertise. Automatic systems help reduce the error in collecting the platelet concentrate with greater precision because they can identify the buffy coat using optical systems. In our opinion, the variability in results reported by some authors may be attributed precisely to the ability to extract the buffy coat accurately during pipetting.

## 4 Conclusion

The scientific literature shows conflicting results, making the data difficult to organize and interpret. For example, while many studies demonstrate significant benefits of using PCs in tissue regeneration (Scarano et al., 2021), others report modest or no results, creating uncertainty about the actual clinical usefulness of these PCs (Franchini et al., 2019). To clarify the concept of heterogeneity in results due to the method used, imagine preparing a dish without specifying the cooking time, temperature, or exact ingredient quantities. Even if the main ingredient is blood, each operator will produce a different dish: some will be perfect, others undercooked or burnt. Without a standard recipe, it is impossible to ensure that the patient always receives the same therapeutic benefit.

Currently, we have numerous devices available to obtain PCs with specific and characteristic features of each method, such as APG, PRF, PRGF, CGF, *etc.* On one hand, this wide range of options offered by companies creates confusion; on the other hand, it allows us to have systems that provide a uniform product for all operators by standardizing centrifugal force, type of vial used, its inclination, and rotation time.

It is also true that there is no consensus on the optimal preparation methods, and a clear and complete categorization of these final products is difficult to find in the literature.

Every small variation in these parameters results in changes in platelet composition, leukocyte profile, and the 3D architecture of fibrin, producing a new and completely different PC.

The positive aspect is that each manual system has a well-defined protocol, which helps clinicians obtain a product that is clearly characterized and studied by various research groups. On the other hand, automatic systems are precise but expensive and bulky to use in a dental office, and current Italian regulations are still unclear as to whether these systems can be used in a dental clinic.

### 4.1 Future research should focus on

Developing universally accepted guidelines for centrifugation parameters, tube types, and activation methods to ensure reproducibility and comparability across studies.

Defining evidence-based criteria for selecting different types of platelet concentrates (e.g., PRF, PRP, CGF) according to specific clinical scenarios such as socket preservation, maxillary sinus augmentation, or periodontal regeneration.

Conducting multicenter randomized controlled clinical trials to clarify the impact of platelet.
